# Comparative Efficacy of Fecal Microbiota Transplantation in Treating Refractory or Recurrent *Clostridioides difficile* Infection among Patients with and without Inflammatory Bowel Disease: A Retrospective Cohort Study

**DOI:** 10.3390/biomedicines12071396

**Published:** 2024-06-23

**Authors:** Jing-Han Chen, Cheng-Hsun Chiu, Chien-Chang Chen, Yi-Ching Chen, Pai-Jui Yeh, Chia-Jung Kuo, Cheng-Tang Chiu, Hao-Tsai Cheng, Yu-Bin Pan, Puo-Hsien Le

**Affiliations:** 1 School of Medicine, Chang Gung University, Taoyuan 333, Taiwan; b07070902@gmail.com; 2 Division of Pediatric Infectious Diseases, Department of Pediatrics, Chang Gung Memorial Hospital, Linkou, Taoyuan 333, Taiwan; chchiu@adm.cgmh.org.tw (C.-H.C.); cemily02@cgmh.org.tw (Y.-C.C.); 3 Chang Gung Microbiota Therapy Center, Chang Gung Memorial Hospital, Linkou, Taoyuan 333, Taiwan; cgj2841@cgmh.org.tw (C.-C.C.); charlie01539@hotmail.com (P.-J.Y.); m7011@cgmh.org.tw (C.-J.K.); ctchiu@cgmh.org.tw (C.-T.C.); 4 Department of Pediatric Gastroenterology, Chang Gung Memorial Hospital, Linkou, Taoyuan 333, Taiwan; 5 Chang Gung Inflammatory Bowel Disease Center, Chang Gung Memorial Hospital, Linkou, Taoyuan 333, Taiwan; 6 Department of Gastroenterology and Hepatology, Chang Gung Memorial Hospital, Linkou, Taoyuan 333, Taiwan; 7 Taiwan Association of the Study of Intestinal Disease (TASID), Taoyuan 333, Taiwan; 8 Division of Gastroenterology and Hepatology, Department of Internal Medicine, New Taipei Municipal Tucheng Hospital, Tucheng, New Taipei City 236, Taiwan; hautai@cgmh.org.tw; 9 Biostatistical Section, Clinical Trial Center, Chang Gung Memorial Hospital, Linkou, Taoyuan 333, Taiwan; jacky1145@cgmh.org.tw

**Keywords:** fecal microbiota transplantation, *Clostridioides difficile* infection, inflammatory bowel disease, refractory CDI, recurrent CDI

## Abstract

*Clostridioides difficile* infection (CDI) worsens inflammatory bowel disease (IBD) prognosis. While fecal microbiota transplantation (FMT) is effective for refractory or recurrent CDI (rrCDI), comparative success rates between IBD and non-IBD patients are scarce. This study addresses this gap. A retrospective cohort study was conducted at Chang Gung Memorial Hospital from April 2019 to October 2023. Patients receiving FMT for rrCDI were categorized into IBD and non-IBD groups. Baseline characteristics and outcomes were compared at one month and one year, with successful FMT defined as the resolution of diarrhea without CDI recurrence. The study included 88 patients: 30 with IBD and 58 without IBD. The IBD group was younger, with fewer comorbidities. Success rates at one month were similar between groups (IBD: 80.0% vs. non-IBD: 78.9%, *p* = 0.908), as were negative toxin tests (IBD: 83.3% vs. non-IBD: 63.8%, *p* = 0.174). One-year success rates (IBD: 70.0% vs. non-IBD: 67.6%, *p* = 0.857) and eradication rates (IBD: 94.4% vs. non-IBD: 73.9%, *p* = 0.112) were also similar. Poor bowel preparation predicted FMT failure at one month (OR = 0.23, *p* = 0.019). No safety issues were reported. FMT is a safe, effective treatment for rrCDI, demonstrating similar success rates in patients with and without IBD.

## 1. Introduction

*Clostridioides difficile* infection (CDI) is a prevalent healthcare-associated infection with a recurrence rate of 15–20% and a mortality rate of 5% [1,2,3]. Its incidence is rising in both adults and children, posing a significant challenge in healthcare settings globally [4,5,6]. Risk factors for CDI include advanced age, hospitalization, certain underlying illnesses like cancer and inflammatory bowel disease (IBD), recent antibiotic usage such as cephalosporins and quinolones [7,8], and the use of proton pump inhibitors (PPIs) [5,9]. IBD is an independent risk factor for CDI, even in the absence of traditional risk factors [10]. In individuals without IBD, the prevalence of CDI is around 0.45% [11]. However, for those with IBD, the risk is significantly higher, with reported incidence rates two to eight times greater than for those without IBD [4,12,13,14,15], particularly among those with ulcerative colitis (UC) [11,16]. The increasing use of advanced therapies, such as biological therapies and small molecules like infliximab and adalimumab, in IBD treatment has led to a rapid increase in CDI prevalence among this population [10,17]. CDI is responsible for about 19% of acute IBD flare-ups [18,19]. Additionally, it leads to poorer clinical outcomes, including a higher risk of IBD therapeutic escalation, readmission, emergency department visits, colectomy, severe disease course, and even mortality [17,20,21,22,23,24,25]. Moreover, treatment failure and recurrence rates are much higher in patients with IBD compared to the general population [10,15].

Fecal microbiota transplantation (FMT) is an effective and safe treatment for refractory or recurrent *Clostridioides difficile* infection (rrCDI) [9,26,27,28,29,30], endorsed by international guidelines [10,15,31,32,33,34,35,36]. In contrast to standard antibiotics, which can exacerbate gut dysbiosis and contribute to CDI, FMT works by reinstating the normal microbial community structure and function in the gut [37]. Therefore, it leads to higher successful rates and lower recurrent rates in treating CDI [9,32]. A network meta-analysis and systematic review of randomized controlled trials have also demonstrated that FMT is the most efficacious among various therapeutic interventions, particularly when compared to commonly used antibiotics such as vancomycin or fidaxomicin [38,39]. Previous studies indicate success rates of 73% for vancomycin and 62.9% for fidaxomicin, with both treatments being associated with a high recurrence rate [40].

FMT is not only effective for the treatment of recurrent CDI in patients without IBD, but also for those with IBD [41,42]. The Rome consensus recommends FMT as a treatment option for both mild and severe recurrent or refractory CDI in patients with IBD [15]. However, previous studies have shown conflicting results regarding the success rate of FMT in treating CDI between patients with and without IBD. Some studies suggest that FMT is less effective in patients with IBD compared to those without IBD [37], while others indicate similar success rates between the two groups [3,41,42]. Due to the lack of short- and long-term data on FMT efficacy (most studies focus on recurrence rates within 1 to 3 months post-transplant), this study aimed to compare the short- and long-term success rates of FMT between both groups and identify predictors of FMT failure to provide valuable insights into personalized treatment strategies for CDI.

## 2. Materials and Methods

### 2.1. Compliance with Ethical Standards

This retrospective study was approved by the Institutional Review Board (IRB) of Chang Gung Medical Foundation (approval No. 202400814B0) under the project titled “Microbiota Transplantation for the Treatment of Refractory or reccurent *Clostridioides difficile* Infection and Prognostic Study”. Due to its retrospective design, the IRB waived the requirement for individual patient consent for the review of medical records.

### 2.2. Patients

In this retrospective cohort analysis, the patients enrolled underwent FMT via colonoscopy for rrCDI at Chang Gung Memorial Hospital in Linkou, Chiayi, and Kaohsiung between April 2019 and October 2023. Participants were divided into two groups based on their underlying conditions: the IBD group and the non-IBD group. The analysis thoroughly investigated risk factors, clinical presentations, and outcomes associated with each group. Donor specimens were obtained from the fecal bank at Chang Gung Microbiota Therapy Center.

### 2.3. Definitions

Successful FMT is defined as the resolution of diarrhea and no recurrence of CDI within one month and one year [3,26,37,43,44]. Recurrence of CDI is characterized by recurrent diarrhea along with laboratory confirmation of positive CD toxin A/B tests [3,10,26,37,44,45,46]. CDI itself is defined by diarrhea (≥3 loose or watery stools per day for at least 2 consecutive days or ≥8 loose stools in 48 h) and a positive stool test for CD toxin [3,26,36,37,44,46]. The Mayo score, a widely employed disease activity index in placebo-controlled trials for UC, consists of four components: rectal bleeding, stool frequency, physician assessment, and endoscopy appearance [9,47]. Each component is assigned a rating from 0 to 3, resulting in a total score ranging from 0 to 12. Mildly active disease is indicated by a score of 3 to 5 points, moderately active disease by a score of 6 to 10 points, and severely active disease by a score of 11 to 12 points. Two shortened versions, namely the partial Mayo score (excluding the endoscopy subscore) and the non-invasive six-point score (comprising only rectal bleeding and stool frequency) [9,48] have been developed and validated.

### 2.4. Data Collection

Comprehensive data, including demographic information (age, gender, BMI), IBD type, Crohn’s Disease Activity Index (CDAI), Mayo score, underlying diseases, antibiotics used for CDI treatment (e.g., Metronidazole, Vancomycin, Fidaxomicin), degree of bowel cleansing, location of FMT transplant, IBD medication (Steroid, Biologics), and associated clinical symptoms (fever, diarrhea, abdominal pain, bloody stools) were meticulously collected from the medical records of qualifying participants. Key laboratory values such as total white blood cell count, hemoglobin, C-reactive protein, and albumin levels were recorded, along with instances of mortality or the date of the last follow-up.

### 2.5. Statistical Analyses

Descriptive statistics were utilized to present continuous variables as mean and standard deviation, and categorical variables as counts or percentages. The Student’s *t*-test was employed for the analysis of continuous variables, while categorical variables were analyzed using either the chi-squared test or Fisher’s exact test, depending on the data distribution. A *p*-value of less than 0.05 was considered statistically significant. To investigate potential predictors for the success rate of FMT, a logistic regression model was applied. First, univariate logistic regression analysis was conducted with all predictors. Then, factors with a *p*-value less than 0.05 were selected for multivariate logistic regression analysis. If only one factor had a *p*-value less than 0.05, all factors with *p*-values less than 0.2 were included in the multivariate logistic regression analysis. All statistical analyses were conducted using SPSS version 26.0 (Armonk, NY, USA, IBM Corp).

## 3. Results

### 3.1. Baseline Characteristics and Clinical Outcome

Our study enrolled 88 patients who underwent FMT via colonoscopy for rrCDI between April 2019 and October 2023. This cohort comprised 30 patients in the IBD group and 58 in the non-IBD group. The primary indications for FMT were refractory CDI in 54 patients, recurrent CDI in 31 patients, and both conditions in 3 patients. Within the IBD subgroup, 20 patients had UC and 10 had CD.

In baseline comparisons, the IBD group was significantly younger (mean ± SD, 45.23 ± 16.45 years vs. 61.90 ± 24.40 years, *p* = 0.001) and had fewer comorbidities, including hypertension (10.0% vs. 55.2%, *p* < 0.001), diabetes mellitus (6.7% vs. 31.0%, *p* = 0.014), and cancer (3.3% vs. 31.0%, *p* = 0.012), compared to the non-IBD group. The majority of FMT procedures (89.8%) were performed in the inpatient setting, and 10.2% were carried out in the outpatient setting. Additionally, the IBD group had lower prior Fidaxomicin use (6.9% vs. 26.3%, *p* = 0.021) and fewer users of HMG-CoA reductase inhibitors (0.0% vs. 15.5%, *p* = 0.025). Detailed baseline characteristics for both IBD and non-IBD groups are outlined in Table 1.

### 3.2. The Clinical Outcomes of rrCDI after FMT

At one-month follow-up, disease severity improved in the IBD group, with the mean partial Mayo score decreasing by 2.9 points, the endoscopic Mayo subscore decreasing by 0.7 points, and CDAI decreasing by 79.98 points. The IBD group exhibited similar negative CD toxin A/B test rates (83.3% vs. 63.8%, *p* = 0.174) and FMT success rates (80.0% vs. 78.9%, *p* = 0.908) compared to the non-IBD group at this time. One year after FMT, the disease activity of the IBD group further improved, with the mean partial Mayo score decreasing by 4.09 points, the endoscopic Mayo subscore decreasing by 0.82 points, and CDAI decreasing by 135.81 points. The eradication rate (94.4% vs. 73.9%, *p* = 0.112) and success rates of FMT (70.0% vs. 67.6%, *p* = 0.857) were comparable between both groups. No safety issues or adverse effects were reported. Success rates of FMT were defined as the absence of CDI recurrence within the follow-up period. Further laboratory findings and clinical outcomes are detailed in Table 2.

### 3.3. Predictors for Successful FMT

One month after FMT, poor bowel preparation was the only negative independent predictor for successful FMT in the multivariate logistic regression analysis (OR = 0.230, 95% CI = 0.067–0.785). During the one-year follow-up, liver cirrhosis and the use of Fidaxomicin were negative independent predictors for successful FMT in the multivariate logistic regression analysis. Liver cirrhosis was associated with a decreased success rate (OR = 0.056, 95% CI = 0.005–0.586), and the use of Fidaxomicin for treating CDI also showed reduced success rates (OR = 0.151, 95% CI = 0.033–0.680). More detailed insights can be found in Table 3 and Table 4.

## 4. Discussion

CDI significantly worsens the prognosis of IBD. However, the treatment of CDI with antibiotics often results in low success rates and high recurrence rates. Our study highlights several critical findings regarding the use of fecal microbiota transplantation (FMT) for treating rrCDI in patients with and without IBD. Despite previous conflicting reports, our data suggest that FMT is equally effective in both patient groups.

In our cohort, the non-IBD group consisted of older patients with a higher prevalence of diabetes mellitus, cancer, and hypertension, which aligns with existing literature indicating a higher risk of CDI in older individuals with comorbidities [3,49]. Previous studies have shown that fidaxomicin has similar efficacy and safety to vancomycin [50]. The higher use of fidaxomicin in the non-IBD group suggests that patients without IBD are more likely to be treated with this second-line antibiotic, whereas IBD patients might prefer FMT due to its potential to improve inflammation concurrently with treating CDI. Although statin use has been associated with severe or complicated CDI in IBD patients [51], the higher rate of HMG-CoA reductase inhibitor use in the non-IBD group in our study can be explained by the older age and higher prevalence of hyperlipidemia and other comorbidities in these patients.

Our results showed no significant difference in the CDI clearance rate and overall success rate of FMT between patients with and without IBD, corroborating findings from the other study [3]. These findings suggest that FMT’s efficacy in treating rrCDI does not significantly differ based on the presence of IBD, despite initial concerns about IBD potentially complicating FMT outcomes. In comparison, another retrospective study found that the effectiveness of FMT in treating recurrent CDI was lower in patients with IBD (CDI clearance rates of 74.4% and 92.1%, respectively; *p* = 0.001) [37]. This discrepancy may be due to differences in study design, patient demographics, and definitions of success.

We observed that poor bowel preparation was a significant predictor of FMT failure at one month ([OR] 0.230, 95% [CI] 0.067–0.785), emphasizing the importance of adequate bowel cleansing before FMT. This finding underscores the need for rigorous pre-FMT preparation protocols to enhance treatment success. Additionally, liver cirrhosis and prior fidaxomicin use emerged as negative predictors for FMT success at one year ([OR] 0.056, 95% [CI] 0.005–0.586 and [OR] 0.151, 95% [CI] 0.033–0.680, respectively). These factors suggest that patients with severe underlying conditions or those who have undergone extensive antibiotic treatment may require additional monitoring and tailored therapeutic approaches.

Our study’s strengths include comprehensive follow-up, providing robust data on the long-term efficacy of FMT in both IBD and non-IBD patients. However, the retrospective design and single-center setting, along with a relatively small sample size, may limit generalizability. Future prospective, multicenter studies are warranted to validate these findings and explore additional factors influencing FMT outcomes.

## 5. Conclusions

FMT via colonoscopy is an effective treatment for rrCDI, demonstrating similar success rates in patients with and without IBD. However, specific patient populations, particularly those with poor bowel preparation, liver cirrhosis, or prior fidaxomicin use, may experience lower success rates. These findings highlight the importance of tailored patient management to optimize FMT outcomes. Further research should focus on refining pre-FMT protocols and identifying additional predictors of success to enhance the efficacy and safety of FMT in diverse patient populations.

## Figures and Tables

**Table 1:** Baseline characteristics of inflammatory bowel disease patients and non-inflammatory bowel disease patients.

Baseline Characteristics				
	Overall (%)	IBD (%)	Non-IBD (%)	*p*-Value
	(n = 88)	(n = 30)	(n = 58)	
Age (years)	56.22 ± 23.31	45.23 ± 16.45	61.90 ± 24.40	0.001 *
Gender, female (%)	36 (40.9)	11 (36.7)	25 (43.1)	0.56
BMI	21.41 ± 4.39	20.88 ± 4.21	21.70 ± 4.49	0.431
IBD type				
Crohn’s disease		10 (33.3)		
CDAI		225.88 ± 126.86		
Ulcerative colitis		20 (66.7)		
Partial Mayo score		5.60 ± 2.78		
Endoscopic Mayo subscore		2.50 ± 0.99		
Underlying diseases				
Cancer	19 (21.6)	1 (3.3)	18 (31.0)	0.002 *
Diabetes mellitus	20 (22.7)	2 (6.7)	18 (31.0)	0.014 *
Hypertension	35 (39.8)	3 (10.0)	32 (55.2)	<0.001 *
Antibiotics used to treat CDI				
Metronidazole	77 (89.5)	26 (89.7)	51 (89.5)	0.979
Vancomycin	42 (48.8)	10 (34.5)	32 (56.1)	0.057
Fidaxomicin	17 (19.8)	2 (6.9)	15 (26.3)	0.044 *
FMT indication				
Refractory CDI	57 (64.8)	17 (56.7)	41 (70.7)	0.208
Recurrent CDI	34 (38.6)	14 (46.7)	20 (34.5)	0.223
Degree of bowel cleansing				
Poor	16 (18.2)	2 (6.7)	14 (24.1)	0.077
Fair	34 (38.6)	15 (50.0)	19 (32.8)	0.115
Good	36 (40.9)	13 (43.3)	23 (39.7)	0.739
Excellent	2 (2.3)	0 (0.0)	2 (3.4)	0.545
Location of transplant				
Terminal ileum	52 (59.8)	23 (79.3)	29 (50.0)	0.009 *
Terminal ileum and cecum	80 (92.0)	28 (32.1)	52 (59.8)	0.265
Others (non-terminal ileum or cecum)	7 (8.0)	1 (1.1)	6 (6.9)	0.265
IBD Medication				
Prednisolone		16 (53.3)		
Biologics		14 (46.7)		
Azathioprine		6 (20.0)		
5-ASA		23 (76.7)		
Additional medication				
Proton Pump Inhibitors	39 (44.3)	10 (33.3)	29 (50.0)	0.136
HMG-CoA reductase inhibitor	9 (10.2)	0 (0.0)	9 (15.5)	0.025 *
Laboratory data at FMT				
CRP (mg/L)	21.12 ± 39.24	20.31 ± 42.60	21.61 ± 37.53	0.702
Albumin (g/dL)	3.53 ± 0.78	3.68 ± 0.59	3.43 ± 0.88	0.234
WBC (1000/μL)	7.94 ± 3.44	8.04 ± 3.21	7.89 ± 3.58	0.845
Hemoglobin (g/dL)	11.32 ± 2.57	11.96 ± 2.78	10.98 ± 2.40	0.098
Duration (first time CDI to FMT), day	108.13 ± 159.91	142.82 ± 220.74	91.38 ± 119.06	0.256

Abbreviations: BMI, body mass index; CDAI, Crohn’s disease activity index; CDI, *Clostridioides difficile* infection; CRP, C-reactive protein; FMT, Fecal microbiota transplant; IBD, inflammatory bowel disease; WBC, white blood cell. * *p* < 0.05.

**Table 2:** Clinical outcomes of *Clostridioides difficile* infection in inflammatory bowel disease patients and non-inflammatory bowel disease patients one month and one year after FMT.

	1 Month				1 Year			
	Overall (%)	IBD (%)	Non-IBD (%)	*p*-Value	Overall (%)	IBD (%)	Non-IBD (%)	*p*-Value
	(n = 88)	(n = 30)	(n = 58)		(n = 88)	(n = 30)	(n = 58)	
BMI	21.20 ± 4.07	20.91 ± 3.81	21.41 ± 4.30	0.33	22.10 ± 4.13	21.75 ± 2.75	22.42 ± 5.17	0.6
BMI change	0.18 ± 1.22	0.10 ± 1.32	0.23 ± 1.17	0.86	1.83 ± 3.40	2.81 ± 4.43	0.89 ± 1.63	0.073
IBD severity								
Partial Mayo score change		−2.90 ± 3.18				−4.09 ± 3.05		
Endoscopic Mayo subscore change		−0.70 ± 0.98				−0.82 ± 1.17		
Mayo score change		−3.80 ± 3.53				−5.00 ± 3.55		
CDAI change		−79.98 ± 58.11				−135.81 ± 75.42		
Therapeutic result								
Success rates of FMT	69 (79.3)	24(80)	45 (78.9)	0.908	37 (68.5)	14 (70)	23 (67.6)	0.857
Negative CD toxin A/B	62 (70.5)	25 (83.3)	37 (63.8)	0.174	34 (82.9)	17 (94.4)	17 (73.9)	0.112
Death	2 (2.6)	0 (0)	2 (4.2)	0.533	6 (8.0)	0 (0)	6 (12.5)	0.082

Abbreviations: BMI, body mass index; CDAI, Crohn’s disease activity index; IBD, inflammatory bowel disease. Definition of successful rate is defined as a resolution of diarrhea and no recurrence of CDI within one month and one year following FMT.

**Table 3:** Predictor for successful FMT at one month: univariate and multivariate logistic regression analysis.

	Univariate Analysis			Multivariate Analysis		
	OR	95%CI	*p*-Value	OR	95%CI	*p*-Value
Age (years)	0.999	0.977–1.002	0.951			
Gender (male)	1.654	0.582–4.700	0.345			
BMI	1.057	0.924–1.21	0.420			
IBD	1.067	0.356–3.198	0.908			
Crohn’s disease	2.550	0.302–21.566	0.390			
CDAI	0.997	0.981–1.013	0.716			
Ulcerative colitis	0.722	0.222–2.349	0.589			
Partial Mayo score	0.928	0.631–1.364	0.704			
Endoscopic Mayo subscore	0.434	0.090–2.085	0.297			
Underlying diseases						
Cancer	0.972	0.279–3.393	0.965			
Chemotherapy	0.464	0.147–1.468	0.191	0.413	0.118–1.447	0.167
Radiotherapy	0.918	0.174–4.853	0.920			
End-stage renal disease	0.308	0.062–1.522	0.148	0.200	0.036–1.120	0.067
Liver cirrhosis	1.619	0.182–14.376	0.665			
Diabetes mellitus	0.722	0.222–2.349	0.589			
Hypertension	0.804	0.282–0.291	0.683			
Fever	1.389	0.355–5.44	0.637			
Antibiotics used to treat CDI						
Metronidazole	0.434	0.051–3.716	0.446			
Vancomycin	1.213	0.426–3.453	0.717			
Fidaxomicin	0.764	0.213–2.732	0.678			
FMT indication						
Refractory CDI	1.408	0.492–4.029	0.524			
Recurrence CDI	0.585	0.203–1.687	0.321			
Patient resource (inpatient)	2.100	0.470–9.373	0.331			
Hospitalization 90 days before FMT	1.081	0.381–3.068	0.884			
Degree of bowel cleansing						
Poor	0.236	0.073–0.766	0.016 *	0.23	0.067–0.785	0.019 *
Fair	1.885	0.605–5.875	0.274			
Good	1.450	0.487–4.314	0.504			
Excellent	-	-	0.999			
Cleaner medication						
PEG	1.184	0.388–3.619	0.767			
Bowklean powder	0.844	0.276–2.581	0.767			
Location of transplant						
Terminal ileum	1.025	0.349–3.015	0.964			
Cecum	0.858	0.281–2.620	0.788			
Others	1.524	0.171–13.574	0.706			
IBD medication						
Prednisolone	1.182	0.197–7.082	0.855			
Biologics	0.357	0.054–2.344	0.283			
Azathioprine	1.316	0.124–13.967	0.820			
5-ASA	0.600	0.058–6.213	0.668			
Additional medication						
Proton Pump Inhibitors	1.730	0.583–5.136	0.324			
HMG-CoA reductase inhibitor	0.903	0.171–4.773	0.905			
Laboratory data at FMT						
CRP (mg/L)	1.004	0.988–1.020	0.648			
Albumin (g/dL)	1.176	0.569–2.432	0.661			
WBC (1000/μL)	0.935	0.806–1.086	0.380			
Hemoglobin (g/dL)	1.132	0.926–1.383	0.226			
Duration (positive CDI to FMT), day	1.001	0.997–1.005	0.629			

Abbreviations: BMI, body mass index; CDAI, Crohn’s disease activity index; CDI, *Clostridioides difficile* infection; CRP, C-reactive protein; FMT, fecal microbiota transplant; IBD, inflammatory bowel disease; WBC, white blood cell. * *p* < 0.05.

**Table 4:** Predictor for successful FMT at one year: univariate and multivariate logistic regression analysis.

	Univariate Analysis			Multivariate Analysis		
	OR	95%CI	*p*-Value	OR	95%CI	*p*-Value
Age (years)	1.012	0.988–1.036	0.339			
Gender (male)	0.868	0.249–3.029	0.824			
BMI	0.995	0.870–1.137	0.939			
IBD	1.116	0.337–3.691	0.857			
Crohn’s disease	3.733	0.421–33.072	0.237			
CDAI	0.993	0.971–1.015	0.513			
Ulcerative colitis	0.560	0.148–2.114	0.392			
Partial Mayo score	0.674	0.372–1.221	0.193			
Endoscopic Mayo subscore	0.391	0.068–2.245	0.293			
Underlying diseases						
Cancer	4.414	0.506–38.526	0.179			
Chemotherapy	1.500	0.350–6.341	0.585			
Radiotherapy	0.914	0.077–10.833	0.943			
End-stage renal disease	0.429	0.055–3.333	0.418			
Liver Cirrhosis	0.090	0.009–0.884	0.039 *	0.056	0.005–0.586	0.016 *
Diabetes mellitus	1.728	0.408–7.315	0.457			
Hypertension	0.604	0.183–1.998	0.409			
Fever						
Antibiotics used to treat CDI						
Metronidazole	0.388	0.042–3.604	0.405			
Vancomycin	0.900	0.283–2.863	0.858			
Fidaxomicin	0.229	0.054–0.966	0.045 *	0.151	0.033–0.680	0.014 *
FMT indication						
Refractory CDI	1.655	0.500–5.470	0.409			
Recurrence CDI	0.604	0.183–1.998	0.409			
Patient resource (inpatient)	0.708	0.068–7.352	0.773			
Hospitalization 90 days before FMT	1.187	0.376–3.751	0.770			
Degree of bowel cleansing						
Poor	1.089	0.244–4.850	0.911			
Fair	0.870	0.269–2.808	0.815			
Good	1.088	0.340–3.488	0.887			
Cleaner medication						
PEG	0.597	0.159–2.224	0.445			
Bowklean powder	1.674	0.446–6.287	0.445			
Location of transplant						
Terminal ileum	1.650	0.519–5.246	0.396			
Cecum	0.376	0.115–1.230	0.106			
IBD medication						
Prednisolone	0.500	0.068–3.675	0.496			
Biologics	6.667	0.609–73.032	0.120			
Azathioprine	0.167	0.019–1.491	0.109			
5-ASA	-	–	0.999			
Additional medication						
Proton Pump Inhibitors	1.066	0.337–3.366	0.914			
HMG-CoA reductase inhibitor	1.172	0.203–6.749	0.859			
Laboratory data at FMT						
CRP (mg/L)	1.015	0.985–1.045	0.330			
Albumin (g/dL)	1.505	0.473–4.794	0.489			
WBC (1000/μL)	0.977	0.816–1.170	0.800			
Hemoglobin (g/dL)	1.041	0.831–1.306	0.726			
Duration (positive CDI to FMT), day	0.995	0.989–1.001	0.102			

Abbreviations: BMI, body mass index; CDAI, Crohn’s disease activity index; CDI, *Clostridioides difficile* infection; CRP, C-reactive protein; FMT, fecal microbiota transplant; IBD, inflammatory bowel disease; WBC, white blood cell. * *p* < 0.05.

## Data Availability

The datasets during and/or analyzed during the current study are available from the corresponding author on reasonable request due to privacy and ethical reasons.
